# Opportunistic Chest CT‐Derived Body Composition for Predicting 90‐Day Adverse Outcomes After Hospitalization for Acute Exacerbation of Chronic Obstructive Pulmonary Disease

**DOI:** 10.1111/crj.70214

**Published:** 2026-07-13

**Authors:** Xueping Lai, Xiaoming Cao

**Affiliations:** ^1^ Department of Pulmonary and Critical Care Medicine Longyan First Affiliated Hospital of Fujian Medical University Longyan Fujian China

**Keywords:** acute exacerbation of chronic obstructive pulmonary disease, body composition, computed tomography, LASSO, machine learning

## Abstract

**Background:**

Patients hospitalized for acute exacerbation of chronic obstructive pulmonary disease (AECOPD) remain at risk of readmission and death after discharge. Opportunistic chest computed tomography (CT) body‐composition metrics may provide additional prognostic information beyond conventional clinical scores.

**Objective:**

To develop and validate a 90‐day adverse‐outcome prediction model for hospitalized AECOPD using admission clinical variables and opportunistic chest CT body‐composition metrics.

**Methods:**

A retrospective modelling cohort of 203 AECOPD admissions from the index centre was analysed. Admissions from 2021 to 2024 formed the development cohort (*n* = 152), and 2025 admissions formed the temporal‐validation cohort (*n* = 51). An external‐validation cohort from another centre included 103 admissions from records screened between 1 January 1 and 1 January 2025 after the model was locked. The primary outcome was 90‐day readmission or death. LASSO was used for variable screening in the development cohort. A feature‐count AUC plateau analysis and prespecified multialgorithm screening were used to lock the final model before validation. DECAF and BAP‐65 were retained as comparator scores.

**Results:**

The 90‐day adverse outcome occurred in 66 of 152 development patients (43.4%), 18 of 51 temporal‐validation patients (35.3%) and 35 of 103 external‐validation patients (34.0%). LASSO retained prior AECOPD admissions, home oxygen before admission, diabetes mellitus, intermuscular adipose tissue area, long‐term NIV before admission, heart rate and coronary artery disease. Feature‐count analysis supported this seven‐predictor set, and multialgorithm screening selected HistGradientBoosting for validation. In temporal validation, the locked model achieved AUC 0.80 (0.64–0.95), sensitivity 0.78 (0.59–0.94), specificity 0.88 (0.76–0.97) and Brier score 0.15 (0.09–0.21), with imperfect calibration (Hosmer–Lemeshow *p* < 0.001). In external validation, the locked model achieved AUC 0.77 (0.66–0.87), sensitivity 0.66 (0.49–0.80), specificity 0.79 (0.71–0.88) and Brier score 0.18 (0.15–0.21); the Hosmer–Lemeshow *p* value was 0.209.

**Conclusions:**

A 90‐day AECOPD prediction model combining clinical and opportunistic CT body‐composition variables showed consistent discrimination across validation cohorts, but calibration remained a key implementation boundary. Formal multicentre validation and calibration updating are needed before routine clinical use.

## Graphical abstract

Using routinely acquired chest CT and admission clinical data, this study developed and temporally validated a model for 90‐day readmission or death after AECOPD hospitalization. Opportunistic CT‐derived body‐composition metrics, especially intermuscular adipose tissue, may add prognostic information beyond conventional clinical scores.

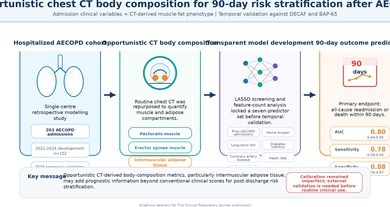

**Abbreviations**
acidaemia: and atrial fibrillation score
AECOPD: acute exacerbation of chronic obstructive pulmonary disease
AUC: area under the receiver operating characteristic curve
BAP‐65: blood urea nitrogen, altered mental status, pulse and age score
COPD: chronic obstructive pulmonary disease
CT: computed tomography
DECAF: dyspnoea
eosinopenia: consolidation
DCA: decision‐curve analysis
HU: Hounsfield unit
ICC: intraclass correlation coefficient
NIV: noninvasive ventilation
SHAP: Shapley additive explanations

## 1 Introduction

Chronic obstructive pulmonary disease (COPD) is a leading cause of morbidity, mortality and health‐system burden worldwide [1]. Acute exacerbations of COPD are clinically important because they accelerate functional decline, precipitate hospitalization and identify patients at high risk for recurrent exacerbation and death. After an AECOPD admission, the period immediately following discharge is vulnerable: Patients may experience residual symptoms, treatment failure, comorbidity destabilization, readmission or death. Current COPD guidance emphasizes integrated assessment of exacerbation history, symptom burden, comorbid disease, gas exchange and care setting when evaluating risk [2].

Established bedside scores such as DECAF and BAP‐65 provide structured risk stratification using clinical and physiological information [3, 4]. These tools are useful comparators for new models, but they do not directly quantify skeletal muscle, adipose tissue distribution or muscle quality. Body‐composition impairment is relevant to COPD because sarcopenia, myosteatosis, malnutrition, inflammation and cardiometabolic disease may reduce physiological reserve and influence postdischarge outcomes.

Chest CT is frequently obtained during AECOPD hospitalization to evaluate pneumonia, pulmonary embolism, malignancy, emphysema phenotype or other cardiothoracic complications. The same examination can be repurposed for opportunistic body‐composition assessment. CT‐based muscle and adipose‐tissue metrics in COPD have been associated with functional status and adverse clinical outcomes, although protocols and anatomical landmarks vary across studies [5, 6]. These observations support the evaluation of CT‐derived body‐composition variables as candidate predictors in 90‐day AECOPD risk modelling.

Prediction‐model studies require a transparent development pathway, clear separation of feature selection from validation, prespecified performance metrics and comparison with existing tools. TRIPOD+AI and PROBAST+AI emphasize reporting of predictors, outcomes, missing‐data handling, model selection, validation, calibration and applicability for regression and machine‐learning prediction models [7, 8]. The present study aimed to develop and validate a 90‐day adverse‐outcome model for hospitalized AECOPD using admission clinical variables and opportunistic chest CT body‐composition metrics, with DECAF and BAP‐65 retained as comparator scores.

## 2 Methods

### 2.1 Study Design, Setting and Participants

This was a retrospective two‐centre modelling and validation study. At the index centre, 365 hospitalized admissions were screened for eligibility; 162 were excluded and 203 AECOPD admissions were included. Index‐centre admissions from 2021 through 2024 were assigned to model development, and 2025 admissions were reserved as a locked temporal‐validation cohort. The external‐validation cohort was drawn from another centre between 1 January 2021 and 1 January 2025. At the external centre, 222 potentially eligible admissions were screened, 119 were excluded, and 103 were included for external validation. The model was finalized before application to the validation cohorts. The source dataset was retained unchanged during analysis.

**FIGURE 1:**
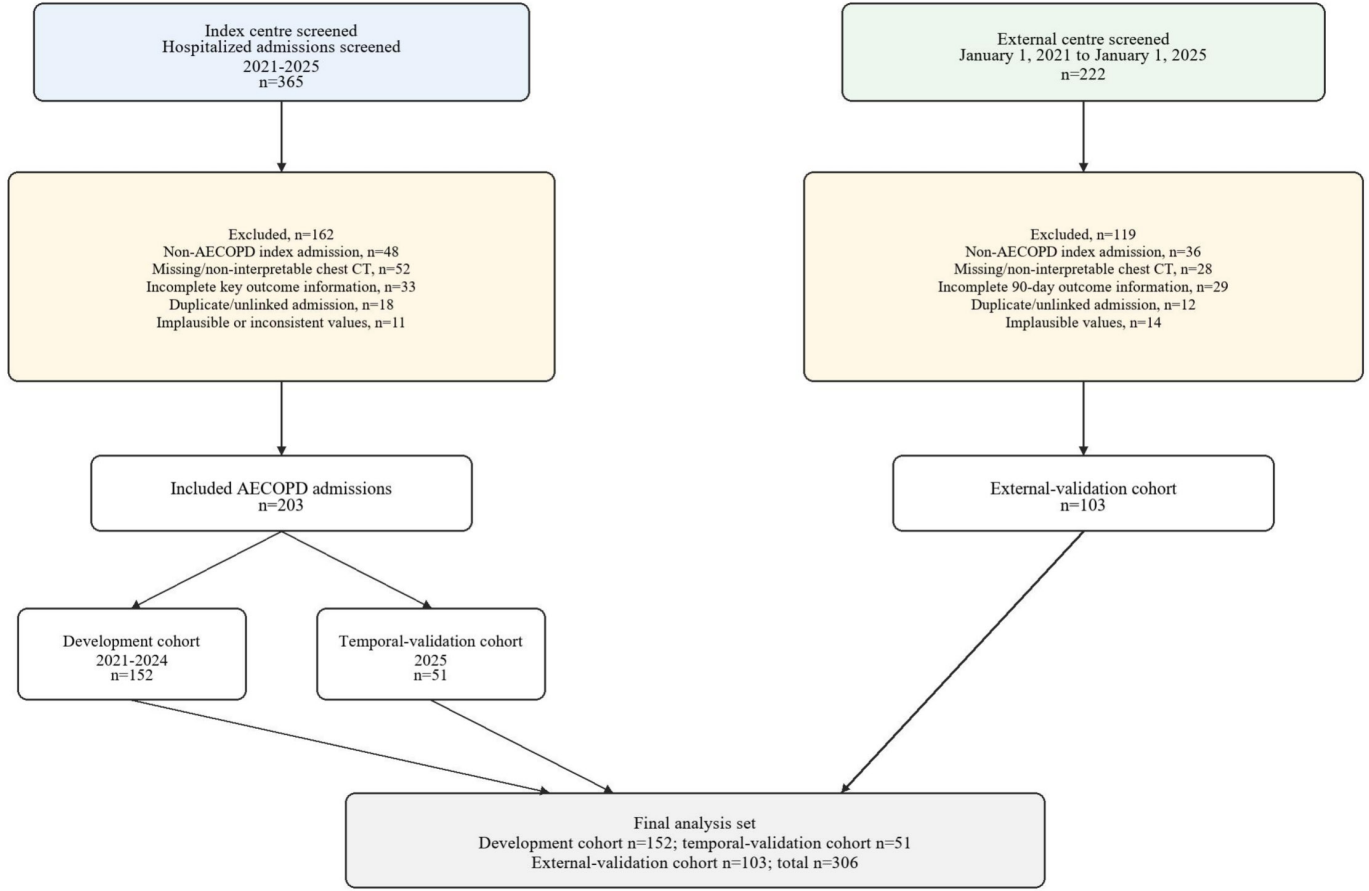
Study flow and validation modelling pathway. The index centre screened 365 hospitalized admissions, excluded 162 records, and included 203 AECOPD admissions, which were split into the development cohort (2021–2024; *n* = 152) and temporal‐validation cohort (2025; *n* = 51). The external centre screened 222 admissions from 1 January 2021 to 1 January 2025; after exclusion of 119 records, 103 admissions were included for external validation.

Eligible records represented adult patients hospitalized for AECOPD with admission clinical variables, laboratory results, arterial blood gas variables, cardiopulmonary imaging information and chest CT body‐composition metrics available for analysis. Inclusion criteria were age 18 years or older, hospitalization for AECOPD during the study period, chest CT performed during or near the index admission, interpretable CT image quality for body‐composition extraction and complete ascertainment of 90‐day readmission and mortality status. Exclusion criteria were a non‐AECOPD index admission, missing or noninterpretable chest CT, incomplete 90‐day outcome information, duplicate admissions that could not be linked to a unique index hospitalization or implausible values after data‐quality review. At the index centre, the corresponding exclusion counts were 48, 52, 33, 18 and 11, respectively; at the external centre, the corresponding exclusion counts were 36, 28, 29, 12 and 14, respectively. Postbaseline treatments and outcome components were not used as candidate predictors. The inclusion flow is shown in Figure 1.

### 2.2 Outcome

The primary endpoint was 90‐day adverse outcome, defined as all‐cause readmission within 90 days after the index AECOPD admission or death within 90 days. Patients with either component were classified as event positive.

### 2.3 CT Acquisition and Body‐Composition Extraction

Chest CT examinations were acquired during the index hospitalization or within 7 days before admission as part of routine clinical care for AECOPD. Examinations were performed using multidetector chest CT scanners with patients in the supine position during end‐inspiration when feasible. Both noncontrast and contrast‐enhanced chest CT examinations were accepted because the target skeletal‐muscle and adipose‐tissue compartments remain segmentable on axial soft‐tissue images when the field of view and image quality are adequate. Images were reconstructed in the axial plane using a standard mediastinal or soft‐tissue kernel, and body‐composition measurements were made on the series with the clearest muscle‐fascia boundary definition. Scans were excluded from image analysis if severe motion artefact, metal artefact, incomplete coverage of the target muscles, truncation of the chest wall or nondiagnostic reconstruction prevented reliable segmentation.

Body‐composition extraction followed a predefined single‐slice thoracic CT protocol implemented in 3D Slicer Version 5.6.2. Pectoralis muscle area was measured on the first axial slice superior to the aortic arch where both pectoralis major and minor muscles were visible. Erector spinae muscle area was measured at the T12 vertebral level on the axial slice with the most complete bilateral erector spinae cross‐section. Skeletal muscle was segmented using the conventional attenuation range of −29 to +150 HU. Intermuscular and subcutaneous adipose‐tissue compartments were segmented using −190 to −30 HU. Pectoralis major and minor were semiautomatically contoured bilaterally, excluding adjacent ribs, vessels, breast tissue and skin. Erector spinae muscles were contoured bilaterally at T12, excluding vertebral bone and paraspinal fat. Intermuscular adipose tissue was defined as adipose‐density tissue within the muscle fascia, whereas subcutaneous adipose tissue was defined as adipose‐density tissue superficial to the outer muscle fascia and deep to the skin surface.

Cross‐sectional areas were recorded in cm^2^. Pectoral muscle index and erector spinae muscle index were calculated by dividing muscle area by height squared and were reported as cm^2^/m^2^. Muscle attenuation was calculated as the mean HU value within the skeletal‐muscle mask and was used as a marker of muscle radiodensity. Segmentations were performed independently by two trained readers who were blinded to the 90‐day outcome, DECAF score, BAP‐65 score and temporal‐validation status. A random subset of 40 examinations was remeasured after a washout interval for reproducibility assessment. Intraobserver and interobserver intraclass correlation coefficients were 0.97 and 0.95 for pectoralis muscle area, 0.96 and 0.94 for erector spinae muscle area, 0.94 and 0.92 for muscle attenuation, 0.93 and 0.91 for intermuscular adipose tissue area and 0.98 and 0.97 for subcutaneous adipose tissue area. Discrepant measurements were resolved by consensus review, and the consensus measurement was used for modelling.

### 2.4 Candidate Predictors and Comparator Scores

Candidate predictors included demographics, smoking exposure, Charlson comorbidity index, cardiovascular and metabolic comorbidities, prior AECOPD admissions, home oxygen use, long‐term NIV before admission, lung function, dyspnoea category, admission vital signs, blood urea nitrogen, eosinophils, albumin, C‐reactive protein, neutrophil‐to‐lymphocyte ratio, arterial pH, PaCO_2_, PaO_2_/FiO_2_ ratio, atrial fibrillation, radiographic consolidation and CT body‐composition metrics. Variables measured after admission treatment decisions or forming the endpoint were excluded from model development. DECAF score, BAP‐65 score and BAP‐65 class were prespecified as comparator scores only and were not entered into feature selection or model training.

### 2.5 Statistical Analysis and Model Development

Continuous variables were summarized uniformly as median with 25th and 75th percentiles. Categorical variables were summarized as *n* (%). Development, temporal‐validation and external‐validation cohorts were compared using Kruskal–Wallis *H* tests for continuous or ordinal variables and Pearson chi‐square tests for categorical variables. Event versus nonevent comparisons were performed in the development cohort only. *p* values were interpreted descriptively and were not used as the primary feature‐selection strategy.

All eligible candidate predictors were entered into LASSO penalized binary‐outcome modelling in the development cohort. Continuous variables were median‐imputed and standardized; categorical variables were imputed using the most frequent category and one‐hot encoded. LASSO tuning used five‐fold cross‐validation. Encoded coefficients were aggregated back to the original clinical variable so that a categorical predictor was not counted as multiple separate variables solely because of one‐hot encoding.

Collinearity review of candidate predictors is reported in Supplementary Table 1. LASSO‐ranked variables were used in feature‐count AUC plateau analysis to identify a compact predictor set for downstream modelling. Candidate algorithms were screened within the development cohort using the selected feature count. The model‐selection hierarchy was prespecified as follows: Algorithms were ranked primarily by cross‐validated AUC; when AUC values were close, lower Brier score and better development‐cohort calibration were used as secondary criteria; sensitivity‐specificity balance and model simplicity were considered as tertiary criteria. The final model was locked before temporal and external validation.

Model performance was evaluated using AUC with bootstrap 95% confidence intervals, sensitivity, specificity, positive predictive value, negative predictive value, Brier score, Hosmer–Lemeshow statistic, calibration curves, decision‐curve analysis and clinical‐impact curves. The development‐cohort Youden threshold was applied to the temporal‐validation and external‐validation cohorts. Calibration was interpreted directly rather than inferred from discrimination. The locked model was compared with DECAF, BAP‐65, BAP‐65 class and important single‐predictor models. SHAP was used for model interpretation. Analyses were performed in Python 3.11 using pandas, scipy, scikit‐learn, statsmodels, matplotlib, shap, joblib, python‐docx, openpyxl and Streamlit.

## 3 Results

### 3.1 Study Population and Event Rate

The analysis included 306 hospitalized AECOPD admissions: 152 admissions in the 2021–2024 development cohort, 51 admissions in the 2025 temporal‐validation cohort and 103 admissions in the external‐validation cohort from another centre (Figure 1 and Table 1). The 90‐day adverse outcome occurred in 66 of 152 development patients (43.4%), 18 of 51 temporal‐validation patients (35.3%) and 35 of 103 external‐validation patients (34.0%). Overall three‐cohort comparison statistics and *p* values are reported in Table 1.

**TABLE 1:** Baseline characteristics across development, temporal‐validation, and external‐validation cohorts.

Variable	Development cohort (*n* = 152)	Temporal‐validation cohort (*n* = 51)	External‐validation cohort (*n* = 103)	Statistic	*p*
90‐Day adverse outcome, *n* (%)	66 (43.42%)	18 (35.29%)	35 (33.98%)	χ ^2^ = 2.64	0.268
Age, years	71.50 (66.00, 76.00)	72.00 (65.50, 77.00)	71.15 (64.71, 77.99)	*H* = 0.06	0.972
Sex: male, *n* (%)	113 (74.34%)	38 (74.51%)	81 (78.64%)	χ ^2^ = 0.68	0.713
Body mass index, kg/m^2^	22.30 (20.00, 24.32)	23.10 (21.20, 24.55)	22.60 (19.68, 24.13)	*H* = 2.71	0.258
Smoking status: current	43 (28.29%)	25 (49.02%)	34 (33.01%)	χ ^2^ = 7.80	0.099
Former	76 (50.00%)	18 (35.29%)	51 (49.51%)		
Never	33 (21.71%)	8 (15.69%)	18 (17.48%)		
Smoking exposure, pack‐years	33.50 (12.50, 43.00)	33.00 (17.00, 49.50)	35.86 (16.04, 46.49)	*H* = 2.81	0.245
Charlson comorbidity index	3.00 (2.00, 4.00)	3.00 (2.00, 4.00)	3.00 (2.00, 4.00)	*H* = 3.72	0.156
AECOPD admissions in the previous 12 months	1.00 (0.00, 2.00)	0.00 (0.00, 1.00)	1.00 (0.00, 2.00)	*H* = 4.90	0.086
Coronary artery disease: yes, *n* (%)	47 (30.92%)	13 (25.49%)	25 (24.27%)	χ ^2^ = 1.51	0.469
Heart failure: yes, *n* (%)	29 (19.08%)	9 (17.65%)	20 (19.42%)	χ ^2^ χ ^2^ = 0.07	0.964
Diabetes mellitus: yes, *n* (%)	36 (23.68%)	12 (23.53%)	22 (21.36%)	χ ^2^ = 0.20	0.904
Chronic kidney disease: yes, *n* (%)	45 (29.61%)	17 (33.33%)	26 (25.24%)	χ ^2^ = 1.20	0.550
Home oxygen before admission: yes, *n* (%)	33 (21.71%)	5 (9.80%)	23 (22.33%)	χ ^2^ = 3.95	0.139
Long‐term NIV before admission: yes, *n* (%)	17 (11.18%)	3 (5.88%)	10 (9.71%)	χ ^2^ = 1.22	0.545
FEV1 percent predicted	34.00 (22.75, 41.00)	38.00 (28.00, 44.50)	32.79 (25.32, 39.90)	*H* = 5.31	0.070
eMRCD dyspnoea category: 0–4	68 (44.74%)	27 (52.94%)	45 (43.69%)	χ ^2^ = 3.77	0.437
5a	55 (36.18%)	12 (23.53%)	40 (38.83%)		
5b	29 (19.08%)	12 (23.53%)	18 (17.48%)		
Respiratory rate, breaths/min	25.00 (23.00, 28.00)	25.00 (23.00, 27.00)	25.00 (23.00, 27.00)	*H* = 0.84	0.658
Heart rate, beats/min	102.50 (91.75, 112.00)	101.00 (91.00, 116.00)	103.42 (92.74, 119.99)	*H* = 1.36	0.507
Blood urea nitrogen, mg/dL	20.20 (14.57, 26.80)	20.20 (13.90, 28.90)	20.05 (14.92, 26.37)	*H* = 0.10	0.953
Albumin, g/L	35.70 (32.70, 38.80)	37.60 (33.55, 39.90)	35.81 (32.71, 39.57)	*H* = 1.80	0.407
C‐reactive protein, mg/L	23.55 (14.72, 33.15)	19.20 (13.50, 24.15)	22.51 (15.45, 33.17)	*H* = 3.56	0.169
Arterial pH	7.37 (7.32, 7.41)	7.38 (7.34, 7.43)	7.38 (7.33, 7.40)	*H* = 2.07	0.356
PaCO_2_, mmHg	51.95 (43.95, 58.12)	49.80 (44.25, 57.60)	54.14 (45.61, 59.77)	*H* = 3.16	0.206
PaO2/FiO_2_ ratio	218.00 (183.00, 262.25)	245.00 (183.50, 287.50)	220.19 (175.86, 270.60)	*H* = 2.86	0.239
Radiographic consolidation: yes, *n* (%)	52 (34.21%)	14 (27.45%)	31 (30.10%)	χ ^2^ = 0.99	0.610
Muscle attenuation, HU	30.50 (28.08, 34.23)	32.20 (28.85, 34.75)	30.96 (27.15, 34.21)	*H* = 1.45	0.485
Intermuscular adipose tissue area, cm^2^	10.70 (8.47, 12.83)	11.30 (7.55, 13.30)	11.30 (8.51, 14.55)	*H* = 2.26	0.322
Subcutaneous adipose tissue area, cm^2^	82.80 (35.17, 112.95)	99.30 (54.20, 128.70)	71.88 (37.91, 110.52)	*H* = 6.17	0.046
Pectoral muscle index, cm^2^/m^2^	12.75 (10.89, 14.26)	13.19 (11.71, 14.46)	12.94 (11.28, 14.17)	*H* = 1.64	0.441
Erector spinae muscle index, cm^2^/m^2^	21.27 (18.57, 23.57)	21.19 (18.42, 23.67)	20.92 (18.68, 22.73)	*H* = 0.19	0.912
DECAF total score	2.00 (1.00, 3.00)	1.00 (0.00, 3.00)	3.00 (2.00, 3.00)	*H* = 48.14	< 0.001
BAP‐65 total score	2.00 (1.00, 2.00)	2.00 (1.00, 2.00)	2.00 (2.00, 3.00)	*H* = 52.94	< 0.001

*Note:* Values are shown as median (25th percentile, 75th percentile) or *n* (%). *H* denotes the Kruskal–Wallis statistic for continuous or ordinal variables; chi‐square denotes the Pearson chi‐square statistic for categorical variables. *p* values compare the three cohorts and are descriptive.

### 3.2 Development‐Cohort Signals

Compared with patients without a 90‐day adverse outcome, those with an event in the development cohort were generally older and had less favourable systemic and body‐composition profiles. In particular, they had a higher burden of prior AECOPD admissions over the previous 12 months and were more likely to be receiving home oxygen before admission. They also showed less favourable laboratory and body‐composition profiles, including higher blood urea nitrogen, lower albumin and greater intermuscular adipose tissue area. Detailed comparisons are presented in Table 2: prior AECOPD admissions, 1.00 (0.00, 1.00) versus 1.00(1.00, 2.00) (*p* < 0.001); home oxygen use, 10 (11.63%) versus 23 (34.85%) (*p* = 0.001); blood urea nitrogen, 18.10 (12.95, 25.40) versus 22.95 (16.47, 29.10) mg/dL (*p* = 0.010); albumin, 36.25 (32.90, 40.05) versus 35.40 (32.32, 37.35) g/L (*p* = 0.008); and intermuscular adipose tissue area, 10.40 (7.45, 12.47) versus 11.40 (9.62, 13.85) cm^2^ (*p* = 0.009). Collinearity review is provided in Supplementary Table 1.

**TABLE 2:** Development event comparison.

Variable	No 90‐day adverse outcome	90‐day adverse outcome	Statistic	*p*
Age, years	69.50 (64.00, 74.75)	73.00 (68.00, 78.00)	−2.6	0.010
Sex: male, *n* (%)	61 (70.93%)	52 (78.79%)	0.83	0.362
Body mass index, kg/m^2^	22.15 (20.42, 24.75)	22.55 (19.75, 24.20)	0.99	0.323
Smoking status: current	22 (25.58%)	21 (31.82%)	0.78	0.675
Former	44 (51.16%)	32 (48.48%)		
Never	20 (23.26%)	13 (19.70%)		
Smoking exposure, pack‐years	32.00 (13.25, 41.75)	36.00 (12.00, 46.00)	2537.5	0.262
Charlson comorbidity index	3.00 (2.00, 4.00)	4.00 (3.00, 5.00)	2228.5	0.021
Coronary artery disease: yes, *n* (%)	22 (25.58%)	25 (37.88%)	2.1	0.147
Heart failure: yes, *n* (%)	13 (15.12%)	16 (24.24%)	1.47	0.226
Diabetes mellitus: yes, *n* (%)	16 (18.60%)	20 (30.30%)	2.22	0.136
Chronic kidney disease: yes, *n* (%)	22 (25.58%)	23 (34.85%)	1.13	0.289
AECOPD admissions in the previous 12 months	1.00 (0.00, 1.00)	1.00 (1.00, 2.00)	1966.0	< 0.001
Home oxygen before admission: yes, *n* (%)	10 (11.63%)	23 (34.85%)	10.52	0.001
Long‐term NIV before admission: yes, *n* (%)	7 (8.14%)	10 (15.15%)	1.21	0.271
FEV1 percent predicted	35.50 (25.25, 41.00)	31.50 (19.00, 40.75)	3250.5	0.125
eMRCD dyspnoea category: 4	48 (55.81%)	20 (30.30%)	13.47	0.001
5a	29 (33.72%)	26 (39.39%)		
5b	9 (10.47%)	20 (30.30%)		
Respiratory rate, breaths/min	25.00 (23.00, 27.75)	26.00 (23.00, 28.00)	−0.81	0.422
Heart rate, beats/min	100.50 (88.50, 108.00)	105.50 (94.25, 115.00)	−2.23	0.027
Systolic blood pressure, mmHg	131.50 (119.00, 140.00)	126.00 (115.25, 138.75)	0.9	0.368
Body temperature, deg. C	36.80 (36.40, 37.20)	36.85 (36.50, 37.27)	−0.91	0.364
Altered mental status: yes, *n* (%)	5 (5.81%)	8 (12.12%)	1.18	0.278
Blood urea nitrogen, mg/dL	18.10 (12.95, 25.40)	22.95 (16.47, 29.10)	2146.0	0.010
Eosinophils, ×10^9/L	0.08 (0.05, 0.14)	0.07 (0.04, 0.11)	3035.5	0.464
Albumin, g/L	36.25 (32.90, 40.05)	35.40 (32.32, 37.35)	2.69	0.008
C‐reactive protein, mg/L	23.60 (13.90, 32.98)	23.05 (15.15, 36.65)	2855.5	0.950
Neutrophil‐to‐lymphocyte ratio	4.95 (3.82, 5.97)	5.45 (3.73, 7.83)	2461.5	0.162
Arterial pH	7.38 (7.33, 7.41)	7.36 (7.31, 7.42)	1.16	0.250
PaCO_2_, mmHg	50.70 (41.82, 57.85)	54.65 (46.02, 59.55)	−1.85	0.066
PaO_2_/FiO_2_ ratio	232.50 (186.25, 266.75)	204.00 (178.25, 252.00)	2.3	0.023
Atrial fibrillation: yes, *n* (%)	22 (25.58%)	17 (25.76%)	0.0	1.000
Radiographic consolidation: yes, *n* (%)	26 (30.23%)	26 (39.39%)	1.02	0.314
Muscle attenuation, HU	31.20 (28.10, 34.60)	30.20 (28.07, 32.53)	1.33	0.184
Intermuscular adipose tissue area, cm^2^	10.40 (7.45, 12.47)	11.40 (9.62, 13.85)	−2.66	0.009
Subcutaneous adipose tissue area, cm^2^	84.15 (37.57, 122.90)	79.60 (32.02, 104.38)	3160.0	0.230
Pectoral muscle index, cm^2^/m^2^	13.02 (10.86, 14.52)	12.28 (11.01, 13.64)	1.5	0.137
Erector spinae muscle index, cm^2^/m^2^	21.77 (19.42, 23.71)	20.54 (17.76, 23.01)	1.98	0.049
DECAF score	1.00 (1.00, 2.00)	2.00 (1.00, 3.00)	1959.0	< 0.001
BAP‐65 score	1.00 (1.00, 2.00)	2.00 (1.00, 2.00)	1861.0	< 0.001
BAP‐65 class	2.00 (2.00, 3.00)	3.00 (2.00, 3.00)	1861.0	< 0.001

### 3.3 LASSO Feature Selection and Feature‐Count Analysis

**FIGURE 2:**
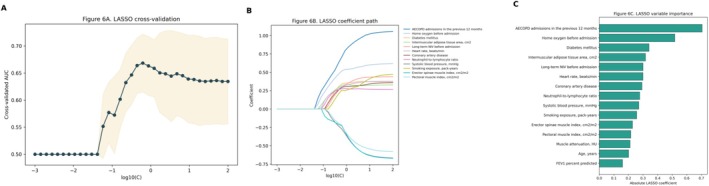
LASSO feature selection for 90‐day adverse outcome. Panel A shows cross‐validated AUC across regularization strengths. Panel B shows the coefficient path. Panel C shows LASSO variable importance aggregated to original predictor names.

LASSO retained the following seven variables for downstream modelling: prior AECOPD admissions, home oxygen before admission, diabetes mellitus, intermuscular adipose tissue area, long‐term NIV before admission, heart rate and coronary artery disease (Figure 2 and Supplementary Table 2). The feature‐count AUC plateau analysis supported the seven‐predictor set used for multialgorithm screening (Supplementary Figure 1).

### 3.4 Model Screening in the Development Cohort

**FIGURE 3:**
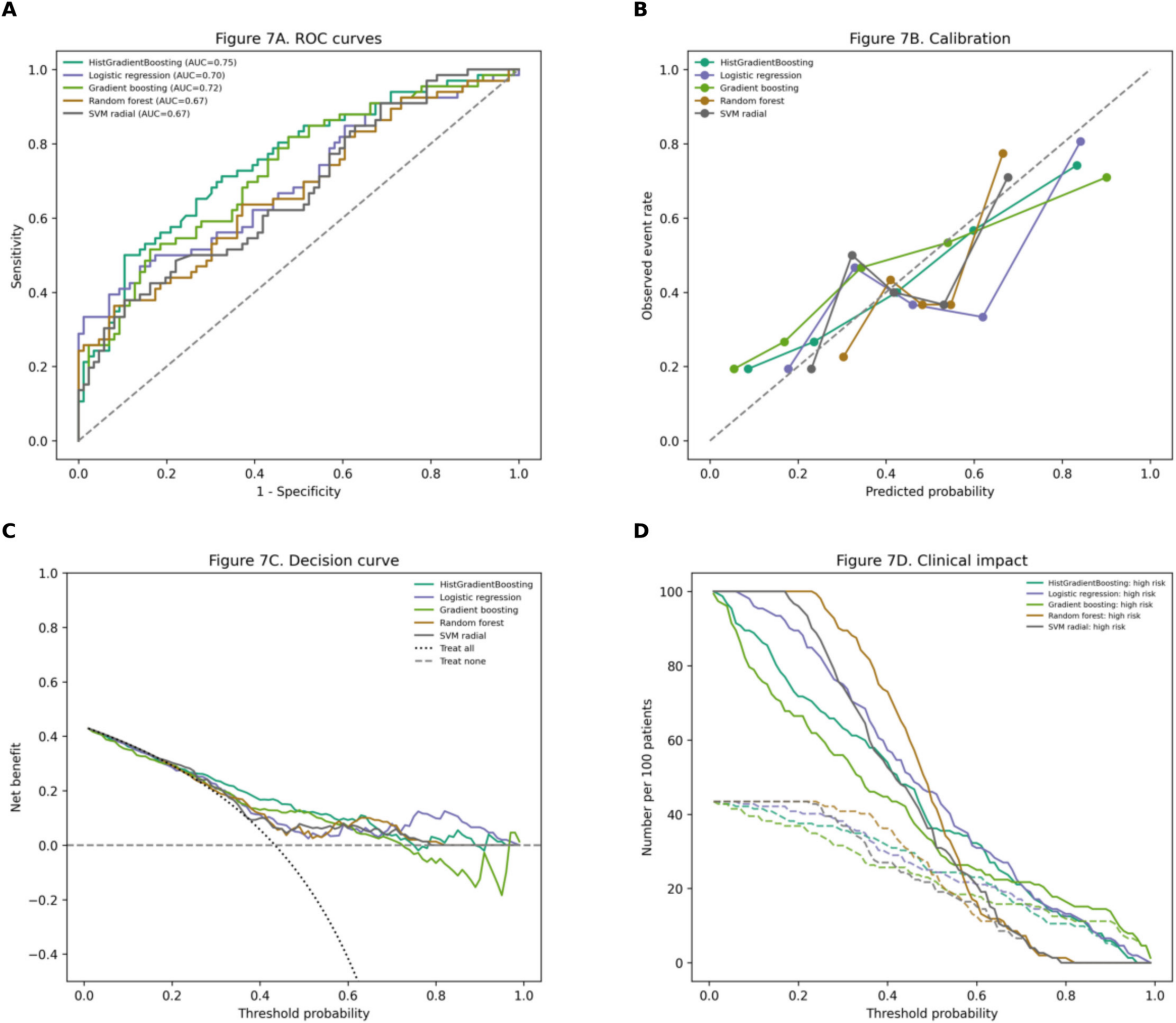
Development‐cohort multialgorithm model screening. Panel A shows ROC curves. Panel B shows calibration curves. Panel C shows decision‐curve analysis. Panel D shows clinical‐impact curves.

In development‐cohort screening, HistGradientBoosting ranked first according to the prespecified model‐selection hierarchy. It achieved a cross‐validated AUC of 0.75, with sensitivity of 0.50, specificity of 0.90 and a Brier score of 0.20 (Figure 3 and Supplementary Table 3).

### 3.5 Temporal and External Validation Performance

When applied to the locked temporal‐validation cohort, the final model achieved an AUC of 0.80 (0.64–0.95), sensitivity of 0.78 (0.59–0.94), specificity of 0.88 (0.76–0.97) and a Brier score of 0.15 (0.09–0.21). The Hosmer–Lemeshow statistic was 28.35 with *p* < 0.001, and temporal calibration was reported separately from discrimination. In the external‐validation cohort, the final model achieved an AUC of 0.77 (0.66–0.87), sensitivity of 0.66 (0.49–0.80), specificity of 0.79 (0.71–0.88) and Brier score of 0.18 (0.15–0.21). External‐validation calibration was not perfect but was less discrepant than the temporal‐validation result (Hosmer–Lemeshow *p* = 0.209).

### 3.6 Comparison With Established Clinical Scores

**FIGURE 4:**
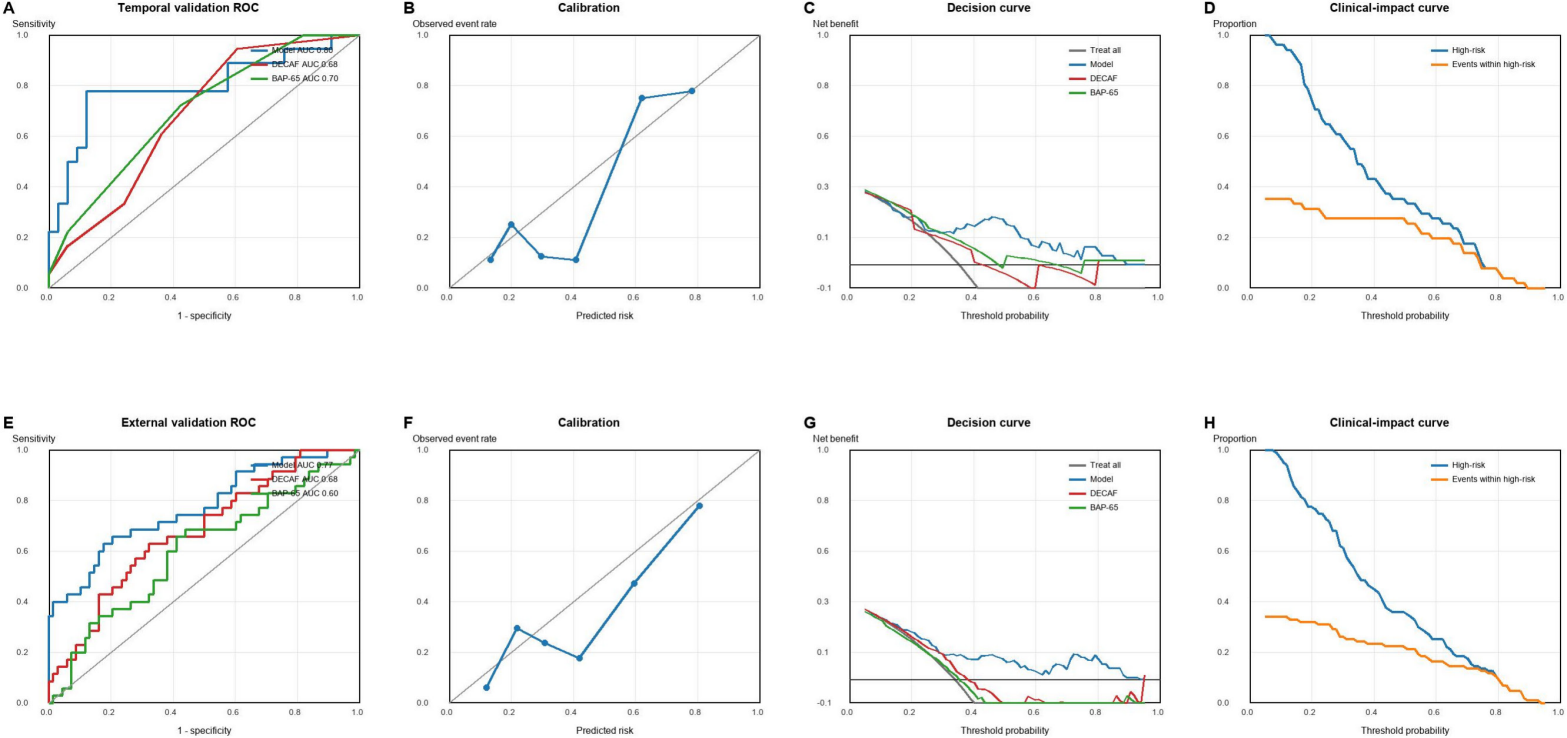
Temporal and external validation performance. Panels A–D show ROC curves, calibration, decision‐curve analysis, and clinical‐impact curves in the temporal‐validation cohort. Panels E–H show the corresponding curves in the external‐validation cohort.

Across the temporal‐validation and external‐validation cohorts, the locked fusion model showed numerically higher discrimination than DECAF and BAP‐65, although confidence intervals still overlapped and calibration findings showed that further recalibration would be required before probability‐based clinical implementation. Detailed comparative results and validation curves are shown in Table 3 and Figure 4.

**TABLE 3:** Validation performance of the locked model and comparator scores.

Cohort	Model	Source	AUC (95% CI)	Sensitivity (95% CI)	Specificity (95% CI)	PPV (95% CI)	NPV (95% CI)	Threshold	HL statistic	HL *p*	Brier score (95% CI)	TP/FP/TN/FN
Temporal validation	Locked fusion model	HistGradientBoosting	0.80 (0.64–0.95)	0.78 (0.59–0.94)	0.88 (0.76–0.97)	0.78 (0.59–0.95)	0.88 (0.76–0.97)	0.47	28.35	< 0.001	0.15 (0.09–0.21)	14/4/29/4
Temporal validation	DECAF score only	decaf_total	0.68 (0.55–0.81)	0.33 (0.13–0.56)	0.76 (0.62–0.89)	0.43 (0.18–0.70)	0.68 (0.53–0.82)	0.64	5.73	0.057	0.22 (0.19–0.26)	6/8/25/12
Temporal validation	BAP‐65 score only	bap65_total	0.70 (0.56–0.83)	0.72 (0.53–0.93)	0.58 (0.39–0.74)	0.48 (0.30–0.68)	0.79 (0.62–0.95)	0.58	4.82	0.090	0.22 (0.18–0.25)	13/14/19/5
External validation	Locked fusion model	HistGradientBoosting	0.77 (0.66–0.87)	0.66 (0.49–0.80)	0.79 (0.71–0.88)	0.62 (0.46–0.77)	0.82 (0.72–0.90)	0.47	10.87	0.209	0.18 (0.15–0.21)	23/14/54/12
External validation	DECAF score only	decaf_total	0.68 (0.59–0.78)	0.74 (0.59–0.87)	0.57 (0.46–0.70)	0.47 (0.34–0.61)	0.81 (0.71–0.91)	0.50	15.73	0.046	0.22 (0.18–0.25)	26/29/39/9
External validation	BAP‐65 score only	bap65_total	0.60 (0.49–0.70)	0.57 (0.42–0.74)	0.57 (0.46–0.69)	0.41 (0.27–0.54)	0.72 (0.61–0.84)	0.50	27.96	< 0.001	0.26 (0.22–0.31)	20/29/39/15

### 3.7 Model Interpretation and Web Calculator

**FIGURE 5:**
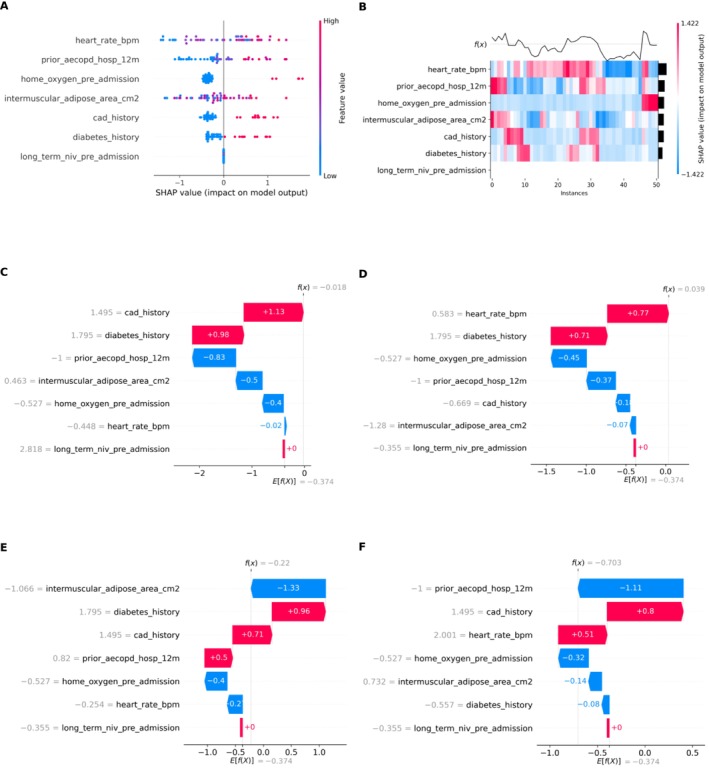
SHAP interpretation of the locked 90‐day model. Panel A shows the beeswarm plot. Panel B shows the SHAP heatmap. Panels C–F show representative true‐positive, false‐positive, true‐negative, and false‐negative waterfall plots.

SHAP analyses were used to visualize the contribution pattern of the retained predictors in the locked model and to illustrate how individual variables influenced model output across patients and representative cases (Figure 5 and Supplementary Figures [Link], [Link]). A 90‐day web‐calculator interface was also prepared to display model inputs, predicted risk and risk‐band output.

## 4 Discussion

This validation‐focused study of hospitalized patients with AECOPD developed a compact 90‐day adverse‐outcome model that integrated routinely available admission variables with opportunistic chest CT body‐composition metrics. The final predictor set converged on four clinically coherent domains: prior disease instability, chronic respiratory support burden, cardiometabolic comorbidity and adverse muscle‐fat phenotype. The locked model showed consistent discrimination across validation cohorts and numerically higher discrimination than DECAF and BAP‐65, suggesting that postdischarge vulnerability after AECOPD may not be fully captured by conventional bedside scores alone. At the same time, calibration remained a central boundary condition, particularly in temporal validation. Taken together, these results support the role of opportunistic CT‐derived body‐composition information as an additional prognostic signal, but the present model is better viewed as a risk‐ranking tool than as a fully deployment‐ready probability model.

Among the retained predictors, prior AECOPD admissions in the previous 12 months are arguably the most intuitively robust. Recurrent exacerbation has long been recognized as a relatively stable susceptibility trait in COPD rather than a chance event, and previous hospitalization consistently emerges as one of the strongest predictors of future readmission and death [9, 10, 11, 12]. This finding is biologically and clinically congruent with the existing literature. Frequent prior admissions likely summarize several adverse processes simultaneously: persistent airway and systemic inflammation, incomplete recovery after previous exacerbations, accumulated frailty, poorer physical function, inhaler or self‐management complexity and heavier comorbidity load. In other words, this variable is not merely a historical marker; it is a compressed summary of disease instability and diminished reserve. Its prominence in our model supports the view that recent exacerbation history remains central even when more sophisticated imaging‐derived variables are introduced.

The combination of home oxygen before admission, long‐term NIV before admission and heart rate also deserves careful interpretation. These variables should not be read as causal drivers of poor outcome; rather, they likely identify a subgroup with chronic respiratory failure, persistent ventilatory burden or more advanced cardiorespiratory vulnerability. In the randomized trial by Murphy and colleagues, home NIV plus oxygen prolonged the time to readmission or death in selected patients with persistent hypercapnia after an acute COPD exacerbation, underscoring that the need for chronic respiratory support is often a marker of severe underlying physiological impairment [13]. Likewise, recent meta‐analytic data indicate that mortality and readmission after exacerbation‐related hospitalization remain high worldwide, with previous hospitalizations and noninvasive ventilation among the strongest adverse prognostic features [14]. Heart rate may contribute a complementary dimension by reflecting sympathetic activation, residual hypoxemia, systemic stress, deconditioning or occult cardiovascular strain. In this respect, the retained predictor pattern in our study is coherent with the broader concept that post‐AECOPD risk is shaped not only by airflow limitation, but also by the severity of chronic ventilatory dependence and the systemic consequences of exacerbation.

Intermuscular adipose tissue area is the most distinctive imaging‐derived signal in the final model and may be the feature that most clearly differentiates this work from conventional score‐based risk stratification. Importantly, a larger intermuscular adipose tissue area should not be interpreted as a favourable nutritional reserve. Rather, it is more consistent with ectopic fat infiltration, myosteatosis and poorer muscle quality. CT‐based work in COPD has shown that higher intermuscular adiposity is associated with increased mortality, whereas higher subcutaneous adiposity may show a different or even opposite relationship [15]. A recent systematic review similarly concluded that CT‐derived muscle and adipose measures are repeatedly associated with clinically relevant COPD outcomes, although anatomical landmarks and analytic protocols remain heterogeneous [5]. Mechanistically, this observation is plausible: myosteatosis may reflect chronic inactivity, insulin resistance, low‐grade systemic inflammation, mitochondrial dysfunction and impaired muscle performance. The direction of association in our study is therefore biologically sensible: The event‐positive group had a larger intermuscular adipose tissue area, suggesting worse muscle quality rather than better nutritional status. This interpretation is further supported by work linking pectoralis muscle attenuation and area to COPD severity, with the broader literature increasingly emphasizing that muscle quality may be as relevant as muscle quantity [16].

The coexistence of diabetes mellitus and coronary artery disease in the final model further supports the view that 90‐day vulnerability after AECOPD is not purely a respiratory phenomenon. Diabetes has been associated with worse short‐term outcomes and higher readmission burden in COPD‐related hospitalizations, likely through a combination of impaired host defence, altered energy metabolism, vascular dysfunction and interaction with obesity or sarcopenic phenotypes [17]. Cardiovascular comorbidity is similarly relevant. COPD and cardiovascular disease share smoking exposure, aging, oxidative stress, systemic inflammation, endothelial dysfunction and autonomic imbalance, and cardiovascular disease in COPD has been linked to worse outcomes including mortality and exacerbation burden [18]. In practical terms, the model's retained variables suggest that 90‐day adverse outcome after AECOPD may be best conceptualized as the result of intersecting respiratory, metabolic, cardiovascular and body‐composition vulnerabilities rather than as a simple extension of the index exacerbation alone.

The clinical value of this approach is to refine postdischarge risk stratification using information that is often already available. DECAF and BAP‐65 remain useful comparators because they are simple, rapid and clinically interpretable [3, 4]. However, they were designed primarily around acute physiological severity and do not explicitly incorporate body‐composition phenotype. Recent AECOPD prediction‐model work and systematic‐review evidence have also emphasized that candidate predictors, endpoints and transportability vary across settings [19, 20]. The model pattern suggests that postdischarge risk may be better captured when recent exacerbation burden and cardiometabolic disease are considered alongside opportunistic CT markers of muscle‐fat quality. This is attractive from an implementation perspective because it does not require a new scan; it repurposes a chest CT that is frequently obtained during admission. If formally validated, such a model could help identify patients who may benefit from intensified discharge planning, earlier follow‐up, pulmonary rehabilitation referral, nutritional and sarcopenia‐oriented assessment, reassessment of oxygen or home NIV needs and closer management of cardiovascular and metabolic comorbidities. At the same time, the present findings also reinforce a key methodological principle from TRIPOD+AI, PROBAST+AI and calibration‐focused prediction research: good discrimination is not enough [7, 8, 21]. Because calibration remained imperfect, the model should not yet be used to trigger fixed probability thresholds for clinical decision‐making. Its current value is stronger for ranking relative risk than for estimating exact absolute risk.

Several limitations merit careful consideration. First, this was a retrospective two‐centre study with a modest temporal‐validation sample, and the number of outcome events in each validation cohort remained limited. This inevitably widens uncertainty around performance estimates and may have contributed to unstable calibration. Second, although the CT segmentation protocol showed high reproducibility, scanner and reconstruction heterogeneity remain potential sources of variation, particularly for attenuation‐based measurements. Third, the composite endpoint of 90‐day readmission or death improves clinical relevance and event capture, but it also combines outcomes that may arise through partially distinct mechanisms. Fourth, the use of multialgorithm screening in a relatively small development cohort introduces a risk of optimism, even though feature selection and model locking were separated from validation. Finally, broader prospective multicentre validation and recalibration are still required before transportability across institutions, scanners, and care pathways can be assumed. Study strengths included a temporally separated validation design, an external‐validation assessment from another centre, clinically interpretable comparator scores, explicit calibration reporting rather than reliance on AUC alone and a reproducible opportunistic CT body‐composition protocol.

In summary, this study showed that 90‐day adverse outcome after hospitalization for AECOPD may be more accurately stratified when conventional admission variables are combined with opportunistic chest CT body‐composition metrics. The retained predictors point to a clinically coherent risk construct centred on prior exacerbation burden, chronic respiratory support requirements, cardiometabolic comorbidity and adverse muscle‐fat phenotype. Although the model showed consistent validation discrimination and numerically outperformed DECAF and BAP‐65, calibration remained an implementation limitation. Prospective multicentre validation, recalibration and assessment of clinical utility are therefore necessary before probability‐based bedside use is recommended.

## Author Contributions

**Xueping Lai:** conceptualization, data curation, formal analysis, investigation, visualization, writing – original draft. **Xiaoming Cao:** conceptualization, methodology, supervision, validation, writing – review and editing, project administration, correspondence. All authors read and approved the final manuscript.

## Funding

The authors have nothing to report.

## Ethics Statement

This retrospective observational modelling study was conducted in accordance with the Declaration of Helsinki and relevant institutional requirements. The study protocol was reviewed and approved by the Ethics Committee of Longyan First Affiliated Hospital of Fujian Medical University. Owing to the retrospective nature of the study and the use of deidentified clinical and imaging data, the requirement for written informed consent was waived by the Ethics Committee.

## Conflicts of Interest

The authors declare no conflicts of interest.

## Supporting information


**Figure S1:** Feature‐count AUC plateau analysis for 90‐day adverse outcome.

**Figure S2:** All‐sample SHAP contribution heatmap for the locked 90‐day model.

**Figure S3:** SHAP dependence plots for the locked 90‐day model.

**Table S1:** Collinearity review of candidate predictors.

**Table S2:** LASSO variable importance for 90‐day adverse outcome.

**Table S3:** Development‐cohort multialgorithm model screening.

## Data Availability

The datasets used and/or analysed during the current study are available from the corresponding author on reasonable request, subject to institutional approval and applicable data‐protection requirements. The code used for model development, validation, figure generation and calculator construction is available from the corresponding author on reasonable request.
