# Supplementary figures and images for: Opportunistic Chest CT‐Derived Body Composition for Predicting 90‐Day Adverse Outcomes After Hospitalization for Acute Exacerbation of Chronic Obstructive Pulmonary Disease
Source: Clin Respir J. 2026 Jul 13;20(7):e70214. doi: 10.1111/crj.70214 (PMC13364507; doi:10.1111/crj.70214)

Supplementary Figure 6. Feature-count AUC plateau

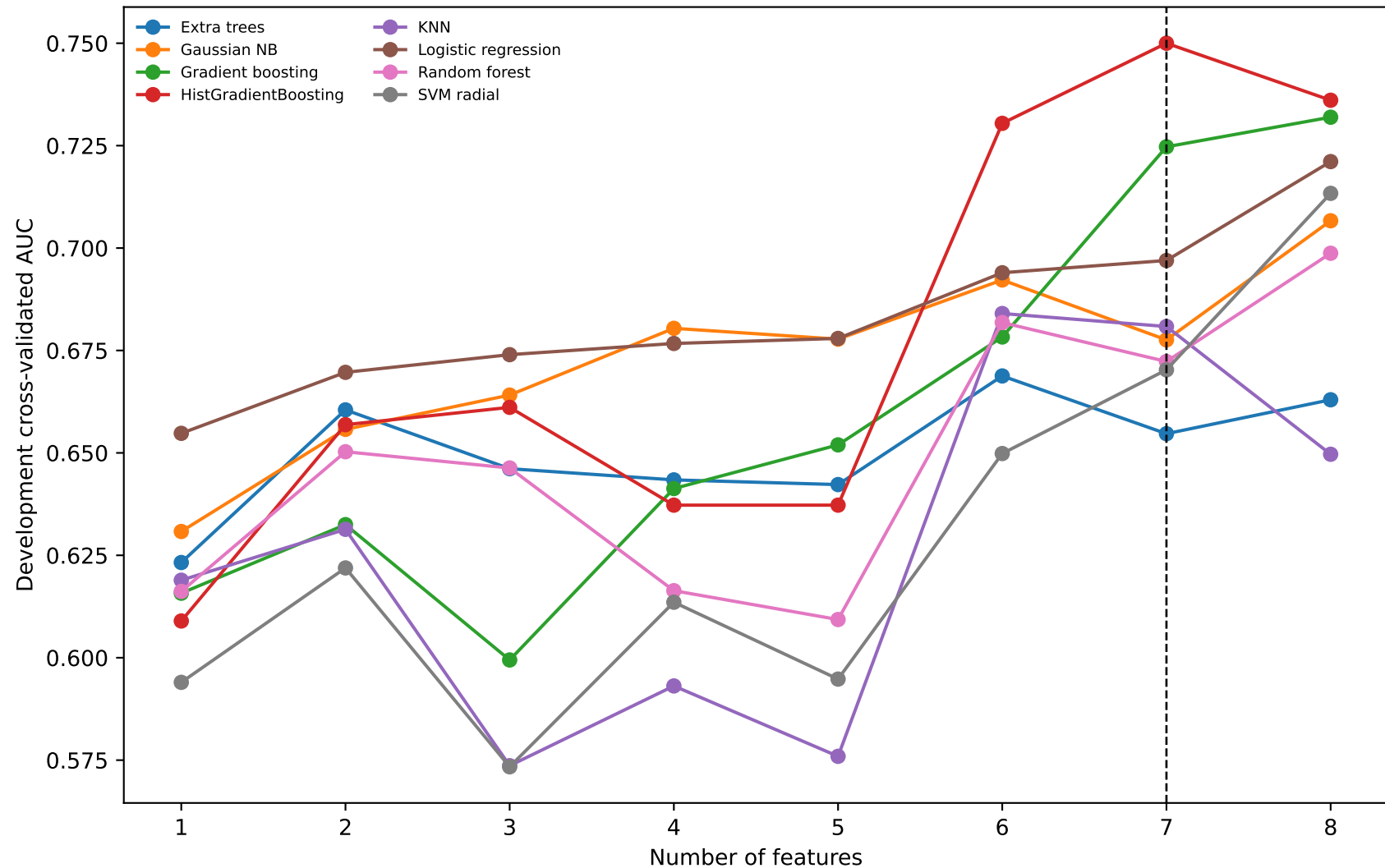

Supplement: Supplementary file 1 — Figure S1: Feature‐count AUC plateau analysis for 90‐day adverse outcome. [file CRJ-20-e70214-s002.pdf]

Supplementary Figure 7. All-sample SHAP contribution heatmap

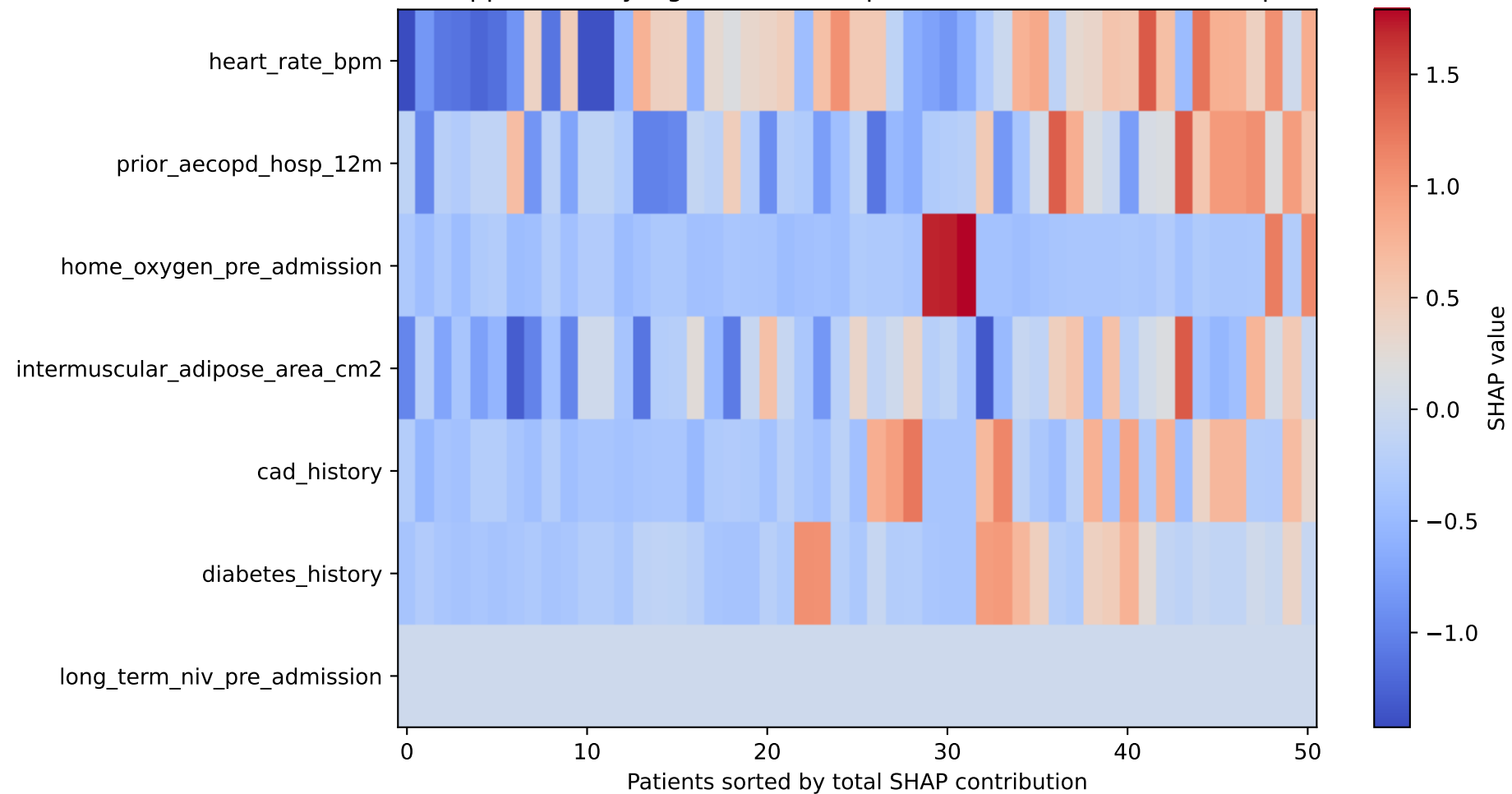

Supplement: Supplementary file 2 — Figure S2: All‐sample SHAP contribution heatmap for the locked 90‐day model. [file CRJ-20-e70214-s003.pdf]

**A**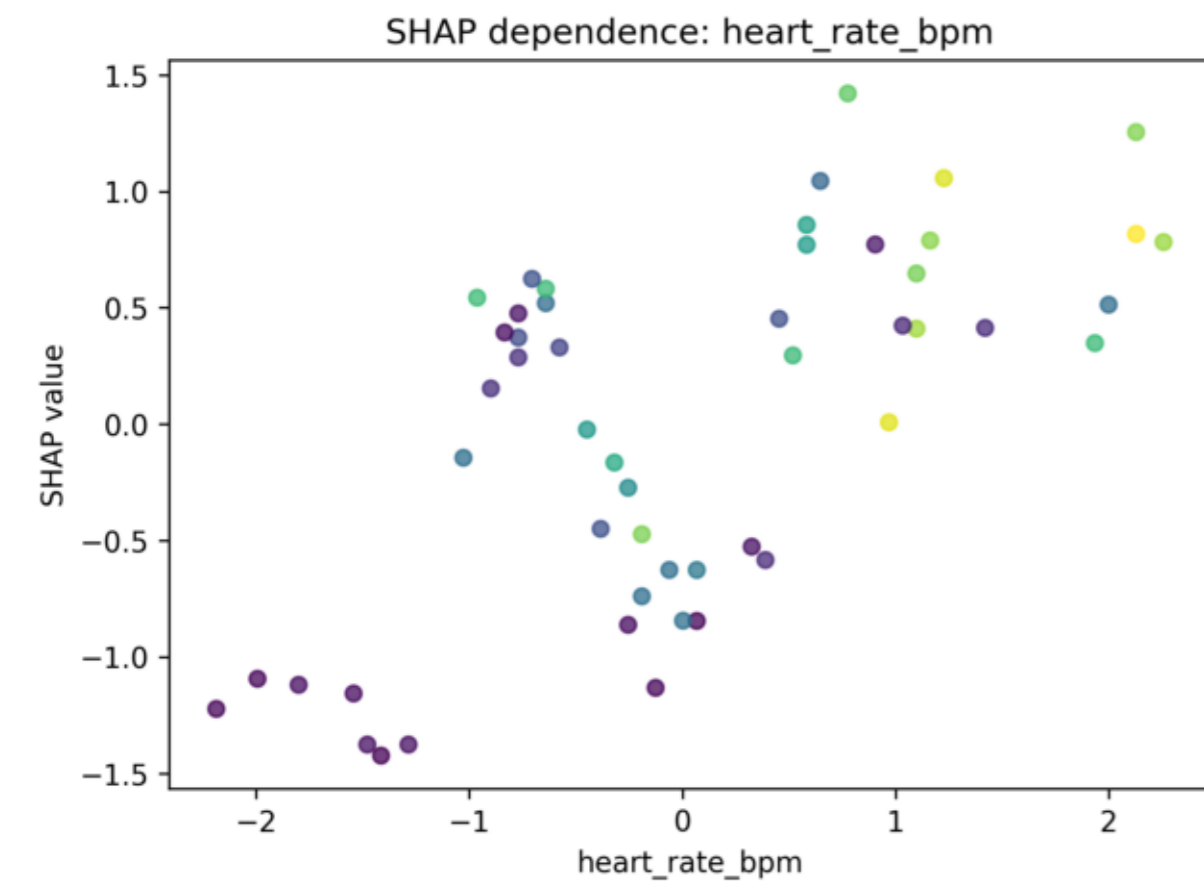**B**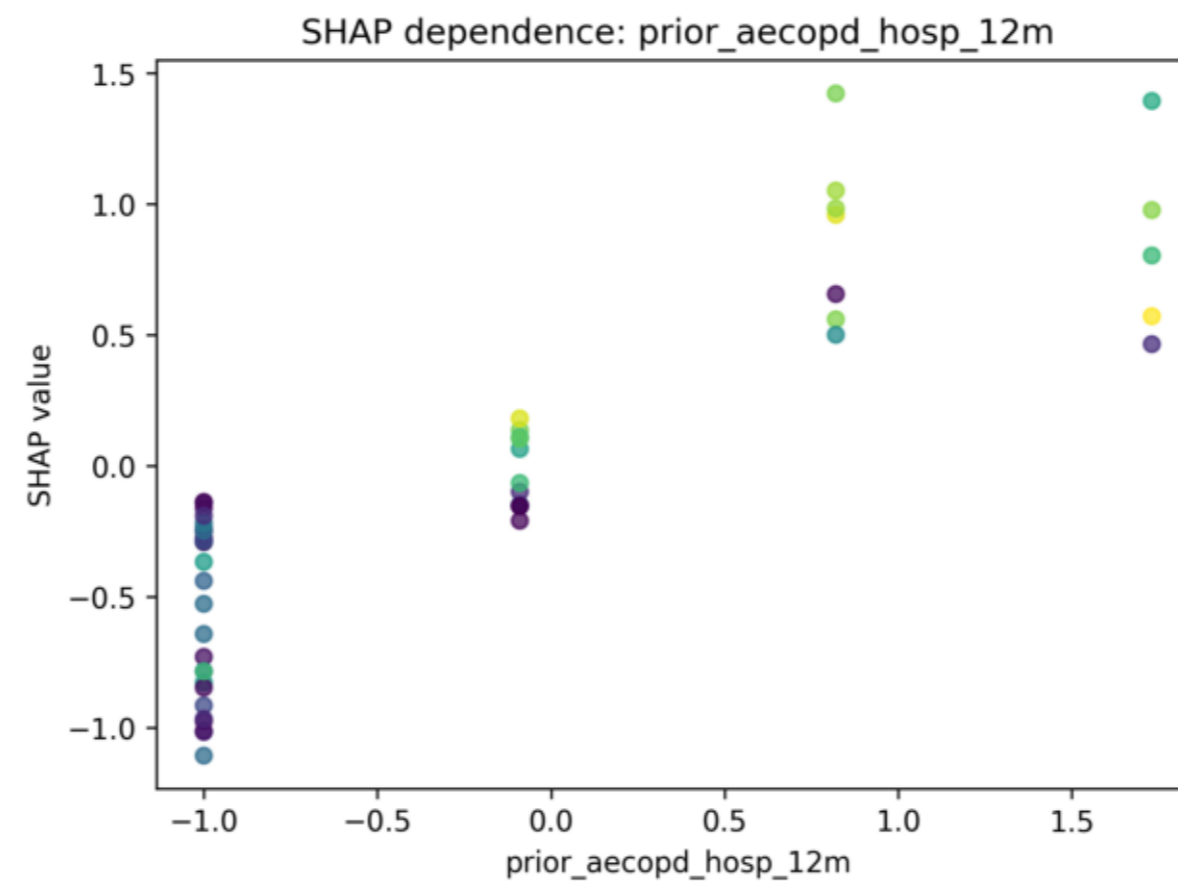**C**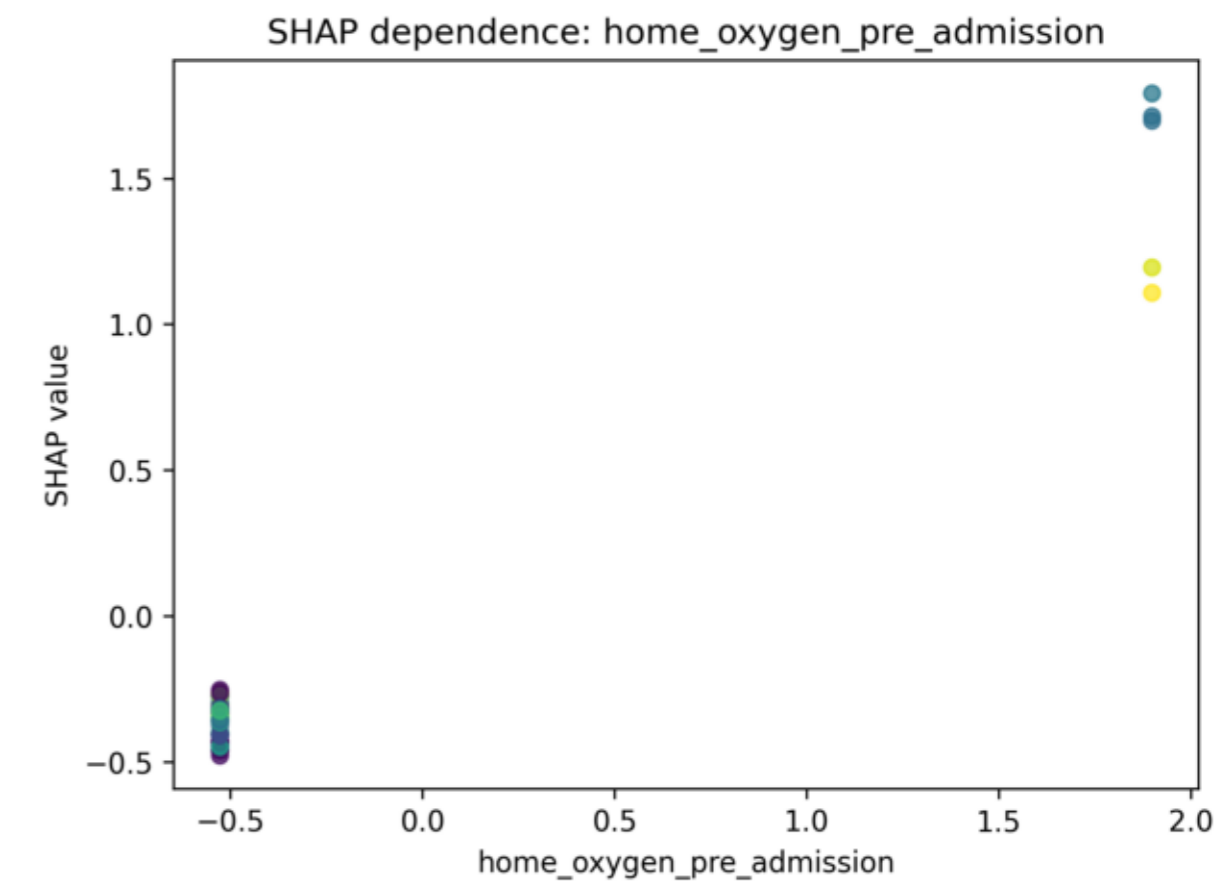**D**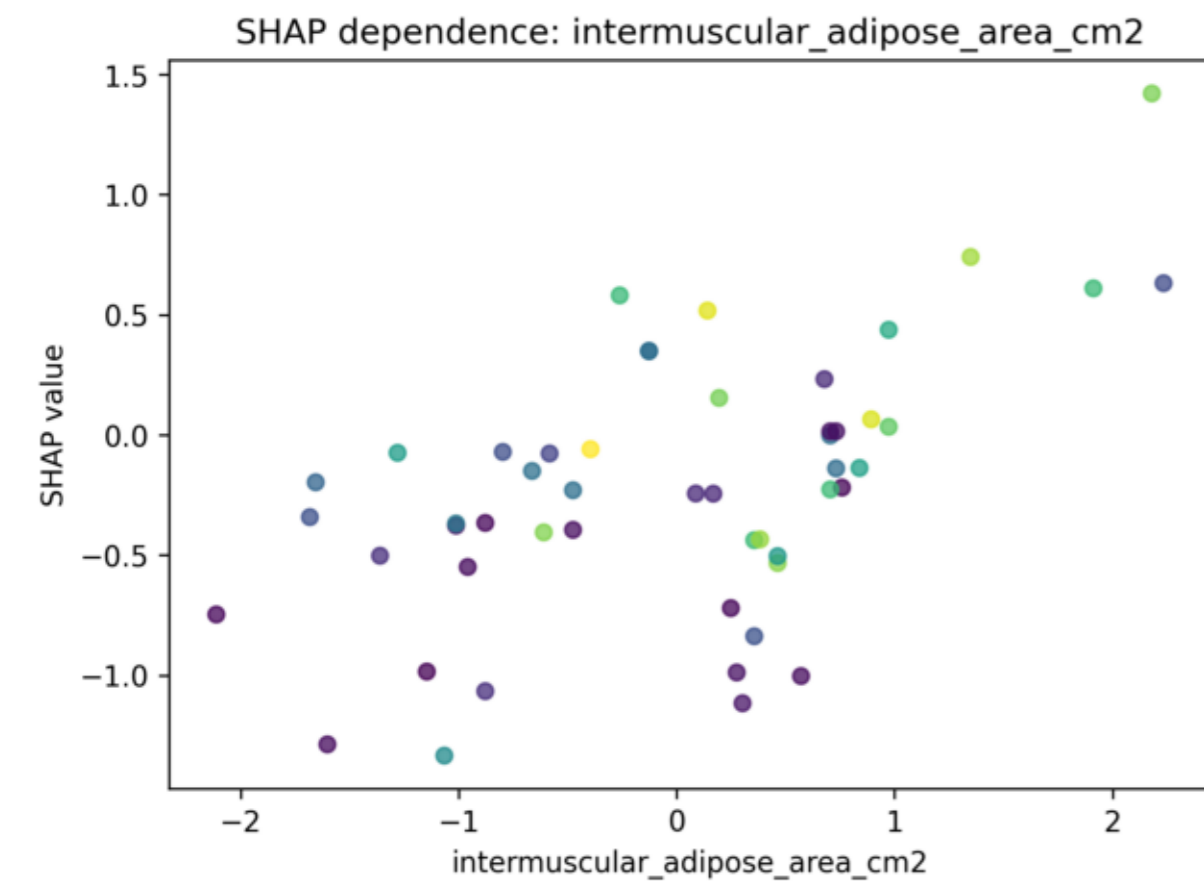**E**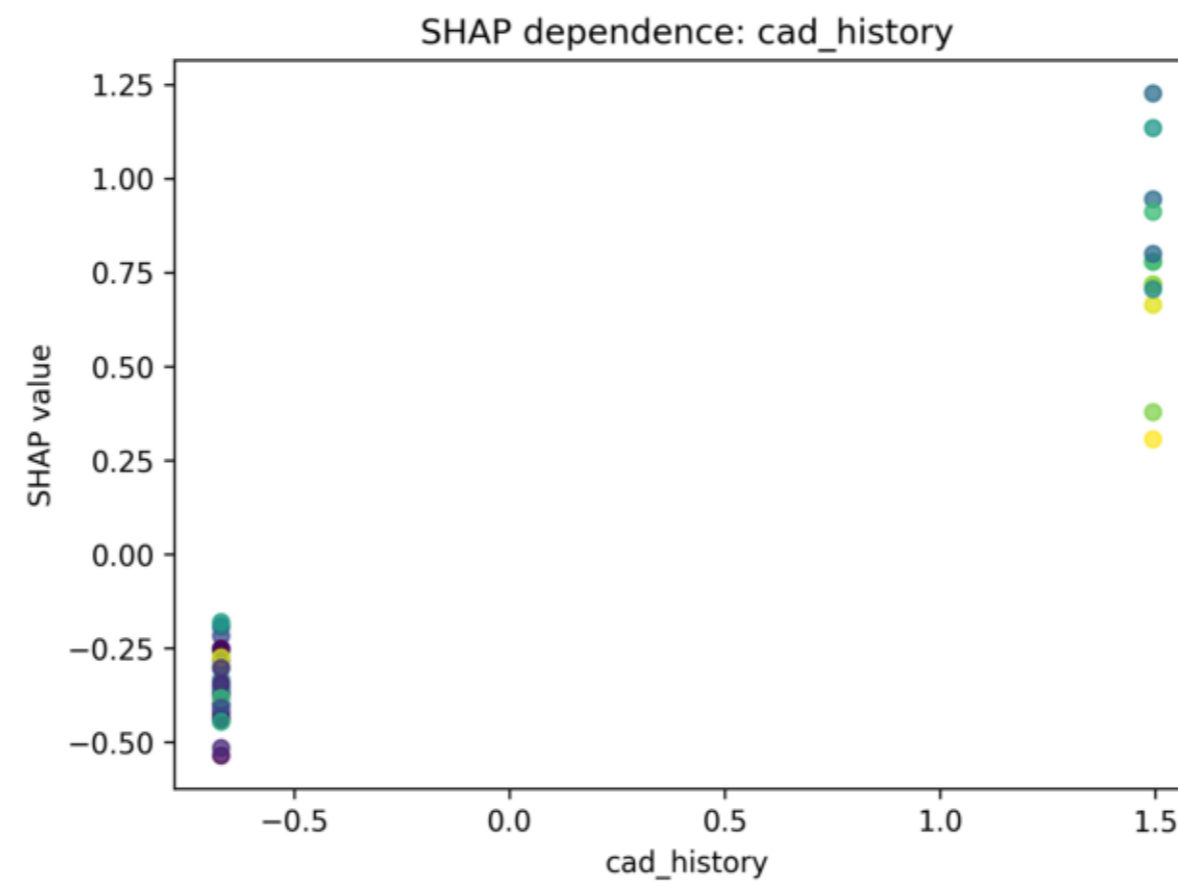**F**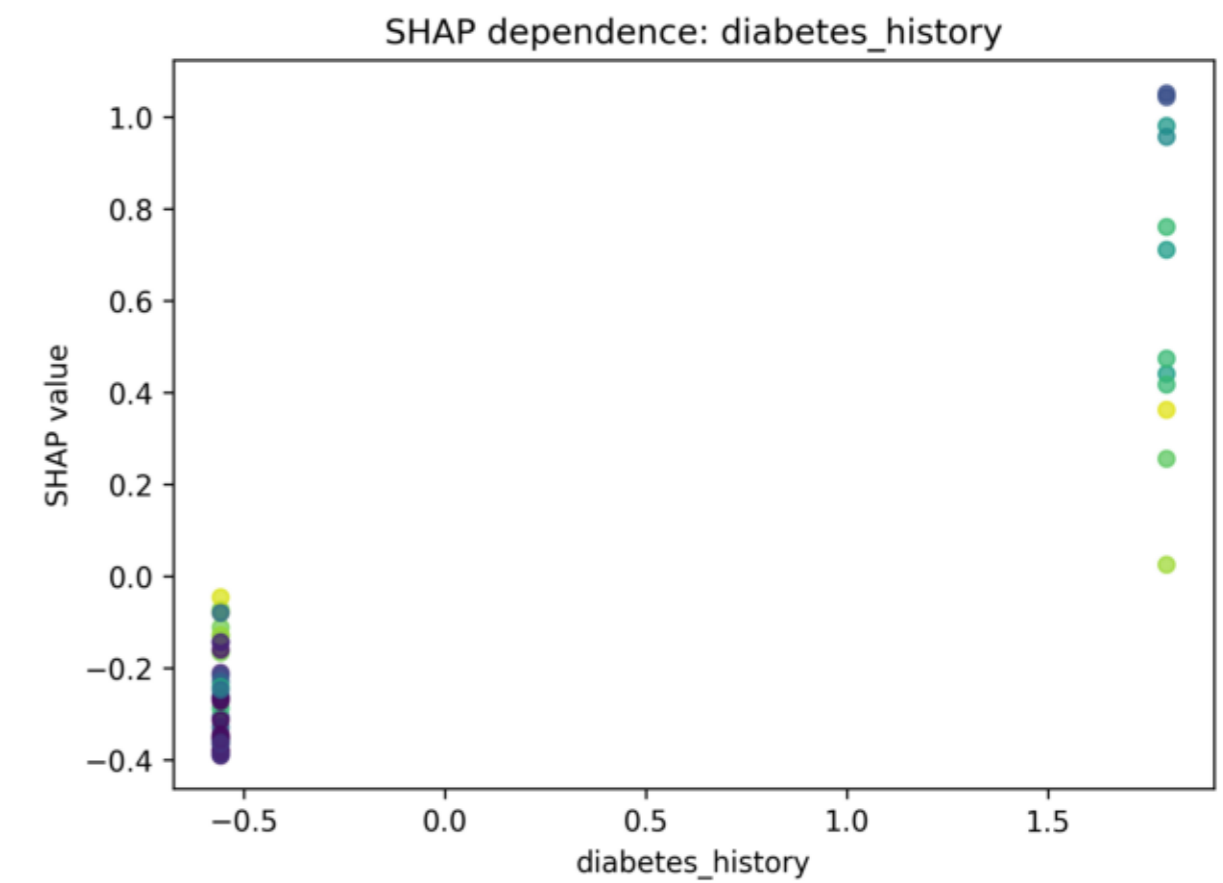

Supplement: Supplementary file 3 — Figure S3: SHAP dependence plots for the locked 90‐day model. [file CRJ-20-e70214-s005.pdf]
