# Supplementary material for: Opportunistic Chest CT‐Derived Body Composition for Predicting 90‐Day Adverse Outcomes After Hospitalization for Acute Exacerbation of Chronic Obstructive Pulmonary Disease
Source: Clin Respir J. 2026 Jul 13;20(7):e70214. doi: 10.1111/crj.70214 (PMC13364507; doi:10.1111/crj.70214)
Supplement: Supplementary file 4 — Table S1: Collinearity review of candidate predictors. [file CRJ-20-e70214-s001.docx]

**Supplementary Table 1. Collinearity review of candidate predictors**

| **Variable** | **Variable type** | **Missing rate (%)** | **Max VIF** | **High pairwise correlation** | **Collinearity flag** | **Decision** |
| --- | --- | --- | --- | --- | --- | --- |
| Age, years | continuous | 0 | 2.16 | No | No | keep |
| Sex | categorical | 0 | 3.66 | No | No | keep |
| Body mass index, kg/m2 | continuous | 0 | 10.32 | Yes | Review | Review for redundancy before feature-count analysis and model screening; retain the clinically interpretable predictor if correlated. |
| Smoking status | categorical | 0 | 3.96 | No | No | keep |
| Smoking exposure, pack-years | continuous | 0 | 3.7 | No | No | keep |
| Charlson comorbidity index | continuous | 0 | 2.23 | No | No | keep |
| Coronary artery disease | binary | 0 | 1.46 | No | No | keep |
| Heart failure | binary | 0 | 1.34 | No | No | keep |
| Diabetes mellitus | binary | 0 | 1.5 | No | No | keep |
| Chronic kidney disease | binary | 0 | 1.54 | No | No | keep |
| AECOPD admissions in the previous 12 months | continuous | 0 | 2.37 | No | No | keep |
| Home oxygen before admission | binary | 0 | 1.51 | No | No | keep |
| Long-term NIV before admission | binary | 0 | 1.33 | No | No | keep |
| FEV1 percent predicted | continuous | 0 | 1.75 | No | No | keep |
| eMRCD dyspnoea category | categorical | 0 | 3.04 | No | No | keep |
| Respiratory rate, breaths/min | continuous | 0 | 1.45 | No | No | keep |
| Heart rate, beats/min | continuous | 0 | 1.47 | No | No | keep |
| Systolic blood pressure, mmHg | continuous | 0 | 1.32 | No | No | keep |
| Body temperature, deg C | continuous | 0 | 1.38 | No | No | keep |
| Altered mental status | binary | 0 | 1.32 | No | No | keep |
| Blood urea nitrogen, mg/dL | continuous | 0 | 1.61 | No | No | keep |
| Eosinophils, x10^9/L | continuous | 0 | 1.3 | No | No | keep |
| Albumin, g/L | continuous | 0 | 1.48 | No | No | keep |
| C-reactive protein, mg/L | continuous | 0 | 1.38 | No | No | keep |
| Neutrophil-to-lymphocyte ratio | continuous | 0 | 1.49 | No | No | keep |
| Arterial pH | continuous | 0 | 2.11 | No | No | keep |
| PaCO2, mmHg | continuous | 0 | 1.39 | No | No | keep |
| PaO2/FiO2 ratio | continuous | 0 | 1.67 | No | No | keep |
| Atrial fibrillation | binary | 0 | 1.62 | No | No | keep |
| Radiographic consolidation | binary | 0 | 1.77 | No | No | keep |
| Muscle attenuation, HU | continuous | 0 | 1.57 | No | No | keep |
| Intermuscular adipose tissue area, cm2 | continuous | 0 | 1.35 | No | No | keep |
| Subcutaneous adipose tissue area, cm2 | continuous | 0 | 9.65 | Yes | Review | Review for redundancy before feature-count analysis and model screening; retain the clinically interpretable predictor if correlated. |
| Pectoral muscle index, cm2/m2 | continuous | 0 | 2.64 | No | No | keep |
| Erector spinae muscle index, cm2/m2 | continuous | 0 | 2.31 | No | No | keep |
