# Supplementary material for: Opportunistic Chest CT‐Derived Body Composition for Predicting 90‐Day Adverse Outcomes After Hospitalization for Acute Exacerbation of Chronic Obstructive Pulmonary Disease
Source: Clin Respir J. 2026 Jul 13;20(7):e70214. doi: 10.1111/crj.70214 (PMC13364507; doi:10.1111/crj.70214)
Supplement: Supplementary file 5 — Table S2: LASSO variable importance for 90‐day adverse outcome. [file CRJ-20-e70214-s004.docx]

**Supplementary Table 7. LASSO variable importance for 90-day adverse outcome.**

| Feature | AbsCoefficient | Coefficient | Variable |
| --- | --- | --- | --- |
| prior_aecopd_hosp_12m | 0.7056549196724201 | 0.7056549196724201 | AECOPD admissions in the previous 12 months |
| home_oxygen_pre_admission | 0.5181175059661719 | 0.5181175059661719 | Home oxygen before admission |
| diabetes_history | 0.34118181854921303 | 0.34118181854921303 | Diabetes mellitus |
| intermuscular_adipose_area_cm2 | 0.31752688243024285 | 0.31752688243024285 | Intermuscular adipose tissue area, cm2 |
| long_term_niv_pre_admission | 0.3005401824870576 | 0.3005401824870576 | Long-term NIV before admission |
| heart_rate_bpm | 0.2996968661375177 | 0.2996968661375177 | Heart rate, beats/min |
| cad_history | 0.29323108414785365 | 0.29323108414785365 | Coronary artery disease |
| nlr | 0.2774095872503929 | 0.2774095872503929 | Neutrophil-to-lymphocyte ratio |
| sbp_mmHg | 0.27134986458975163 | -0.27134986458975163 | Systolic blood pressure, mmHg |
| pack_years | 0.2569619323444533 | 0.2569619323444533 | Smoking exposure, pack-years |
| esmi_cm2_m2 | 0.22828988192589955 | -0.22828988192589955 | Erector spinae muscle index, cm2/m2 |
| pmai_cm2_m2 | 0.21680636871816245 | -0.21680636871816245 | Pectoral muscle index, cm2/m2 |
| muscle_attenuation_hu | 0.21150781519451503 | -0.21150781519451503 | Muscle attenuation, HU |
| age_years | 0.2016666049951339 | 0.2016666049951339 | Age, years |
| fev1_percent_pred | 0.15993501228273965 | 0.15993501228273965 | FEV1 percent predicted |
| eosinophils_x10e9_L | 0.13349293816119706 | -0.13349293816119706 | Eosinophils, x10^9/L |
| bun_mg_dL | 0.12918838967739657 | 0.12918838967739657 | Blood urea nitrogen, mg/dL |
| subcutaneous_adipose_area_cm2 | 0.11594720631075846 | -0.11594720631075846 | Subcutaneous adipose tissue area, cm2 |
| pao2_fio2 | 0.1153759003546395 | -0.1153759003546395 | PaO2/FiO2 ratio |
| resp_rate_bpm | 0.11534255689154502 | 0.11534255689154502 | Respiratory rate, breaths/min |
| bmi_kg_m2 | 0.10767172505661851 | -0.10767172505661851 | Body mass index, kg/m2 |
| albumin_g_L | 0.08906988187211061 | -0.08906988187211061 | Albumin, g/L |
| altered_mental_status | 0.07784254117180024 | 0.07784254117180024 | Altered mental status |
| charlson_comorbidity_index | 0.022442121176240108 | 0.022442121176240108 | Charlson comorbidity index |
| heart_failure_history | 0.010270344670897852 | 0.010270344670897852 | Heart failure |
