# Supplementary material for: Opportunistic Chest CT‐Derived Body Composition for Predicting 90‐Day Adverse Outcomes After Hospitalization for Acute Exacerbation of Chronic Obstructive Pulmonary Disease
Source: Clin Respir J. 2026 Jul 13;20(7):e70214. doi: 10.1111/crj.70214 (PMC13364507; doi:10.1111/crj.70214)
Supplement: Supplementary file 6 — Table S3: Development‐cohort multialgorithm model screening. [file CRJ-20-e70214-s006.docx]

**Supplementary Table 3. Development-cohort multi-algorithm model screening**

| **Selection rank** | **Model** | **AUC** | **Sensitivity** | **Specificity** | **PPV** | **NPV** | **Optimal threshold** | **HL statistic** | **HL P value** | **Brier score** | **Balance** | **Selection rule** |
| --- | --- | --- | --- | --- | --- | --- | --- | --- | --- | --- | --- | --- |
| 1 | HistGradientBoosting | 0.7500000000000001 | 0.5 | 0.8953488372093024 | 0.7857142857142857 | 0.7 | 0.6268427481722353 | 8.94205842198255 | 0.3472074208825393 | 0.2018946566127154 | 0.6046511627906976 | Selected: highest development AUC; lowest Brier score; acceptable development calibration |
| 2 | Gradient boosting | 0.7247181113460184 | 0.5151515151515151 | 0.8372093023255814 | 0.7083333333333334 | 0.6923076923076923 | 0.5058443074162344 | 33.79703198070956 | 4.421459759196278e-05 | 0.2247606569978217 | 0.6779422128259337 | Ranked by AUC first, then Brier score and calibration |
| 3 | Logistic regression | 0.696969696969697 | 0.4696969696969697 | 0.8604651162790697 | 0.7209302325581395 | 0.6788990825688074 | 0.6434523936603069 | 21.7283007494881 | 0.005444851233330161 | 0.2184465982036179 | 0.6092318534179 | Ranked by AUC first, then Brier score and calibration |
| 4 | KNN | 0.6808491895701198 | 0.2575757575757576 | 0.9883720930232558 | 0.9444444444444444 | 0.6343283582089553 | 0.6666666666666666 | 17.00579574799761 | 0.004488818289475338 | 0.2201916829109812 | 0.2692036645525018 | Ranked by AUC first, then Brier score and calibration |
| 5 | Gaussian NB | 0.6775898520084567 | 0.4090909090909091 | 0.8953488372093024 | 0.75 | 0.6637931034482759 | 0.7890214985710984 | 129.7778983729022 | 0.0 | 0.2494671765946653 | 0.5137420718816068 | Ranked by AUC first, then Brier score and calibration |
| 6 | Random forest | 0.6723044397463003 | 0.3636363636363636 | 0.9186046511627907 | 0.7741935483870968 | 0.6528925619834711 | 0.579807906000267 | 16.79588729354787 | 0.03230607437826905 | 0.2216530924269524 | 0.445031712473573 | Ranked by AUC first, then Brier score and calibration |
| 7 | SVM radial | 0.670278365045807 | 0.3787878787878788 | 0.8953488372093024 | 0.7352941176470589 | 0.652542372881356 | 0.5961211609528596 | 12.13922668931099 | 0.1450992007121275 | 0.2223162220179084 | 0.4834390415785764 | Ranked by AUC first, then Brier score and calibration |
| 8 | Extra trees | 0.6546863988724454 | 0.3484848484848485 | 0.9302325581395349 | 0.7931034482758621 | 0.6504065040650406 | 0.587263183497503 | 18.93736600465512 | 0.01519822736809417 | 0.2271995318450873 | 0.4182522903453136 | Ranked by AUC first, then Brier score and calibration |
